# The molecular structure of *Schistosoma mansoni* PNP isoform 2 provides insights into the nucleoside selectivity of PNPs

**DOI:** 10.1371/journal.pone.0203532

**Published:** 2018-09-07

**Authors:** Juliana Roberta Torini, Larissa Romanello, Fernanda Aparecida Heleno Batista, Vitor Hugo Balasco Serrão, Muhammad Faheem, Ana Eliza Zeraik, Louise Bird, Joanne Nettleship, Yamini Reddivari, Ray Owens, Ricardo DeMarco, Júlio César Borges, José Brandão-Neto, Humberto D’Muniz Pereira

**Affiliations:** 1 Laboratório de Biologia Estrutural, Instituto de Física de São Carlos, Universidade de São Paulo, São Carlos, São Paulo, Brazil; 2 Instituto de Química de São Carlos, Universidade de São Paulo - USP, São Carlos, São Paulo, Brazil; 3 Centro Nacional de Pesquisa em Energia e Materiais, Laboratório Nacional de Biociências, Campinas, São Paulo, Brazil; 4 Programa de Pós Graduação em Ciências Genômicas e Biotecnologia, Universidade Católica de Brasília, Brasília, Federal District, Brazil; 5 Laboratório de Biofísica Molecular, Departamento de Biologia Celular, Universidade de Brasília, Brasília, Federal District, Brazil; 6 OPPF-UK, Research Complex at Harwell, Rutherford Appleton Laboratory, Oxford, United Kingdom; 7 Diamond Light Source, Harwell Science and Innovation Campus, Didcot, Oxfordshire, United Kingdom; Universidade Guarulhos, BRAZIL

## Abstract

Purine nucleoside phosphorylases (PNPs) play an important role in the blood fluke parasite *Schistosoma mansoni* as a key enzyme of the purine salvage pathway. Here we present the structural and kinetic characterization of a new PNP isoform from *S*. *mansoni*, *Sm*PNP2. Thermofluorescence screening of different ligands suggested cytidine and cytosine are potential ligands. The binding of cytosine and cytidine were confirmed by isothermal titration calorimetry, with a *K*_D_ of 27 μM for cytosine, and a *K*_M_ of 76.3 μM for cytidine. *Sm*PNP2 also displays catalytic activity against inosine and adenosine, making it the first described PNP with robust catalytic activity towards both pyrimidines and purines. Crystal structures of *Sm*PNP2 with different ligands were obtained and comparison of these structures with the previously described *S*. *mansoni* PNP (*Sm*PNP1) provided clues for the unique capacity of *Sm*PNP2 to bind pyrimidines. When compared with the structure of *Sm*PNP1, substitutions in the vicinity of *Sm*PNP2 active site alter the architecture of the nucleoside base binding site thus permitting an alternative binding mode for nucleosides, with a 180° rotation from the canonical binding mode. The remarkable plasticity of this binding site enhances our understanding of the correlation between structure and nucleotide selectivity, thus suggesting new ways to analyse PNP activity.

## Introduction

Schistosomiasis is a tropical neglected disease that affects almost 206 million people worldwide according to World Health Organization (WHO), causing approximately 250,000 deaths annually [1]. The disease is caused by a blood fluke of genus *Schistosoma* called *Schistosoma mansoni*, which is one of the main species commonly found in Africa, Middle East and the Americas.

It has been described previously that the *de novo* purine synthesis pathway is absent in the adult and schistosomula life stages of *S*. *mansoni*, and they depend exclusively on the purine salvage pathway [2–10]. El Kouni and Naguib [11] investigated the pyrimidine salvage pathway “*in vivo*” and in extracts of *S*. *mansoni*. In the intact worms, cytidine was the most efficiently incorporated pyrimidine precursor, while cytosine has failed to incorporate. The authors were unable to detect cytosine deamination to uracil and the cleavage of cytidine to cytosine.

Purine nucleoside phosphorylase (PNP) (E.C 2.4.2.1) is an enzyme responsible for reversible phosphorolysis of purine nucleosides, generating ribose-1-phosphate and their corresponding bases. PNPs belong to the Nucleoside phosphorylase (NP-I) super-family and are classified into two groups: a Low Molecular Weight (LMW) group, comprised of homotrimers with specificity for inosine, guanosine and their analogs and High Molecular Weight (HMW) group, that are homohexamers with a broader specificity accepting both 6-oxo and 6-amino purine nucleosides as well as many other analogues [12]. Other members of the NP-I family are the pyrimidine phosphorylases, for which Uridine Phosphorylase is the best-known example [13].

Recently Zhou et al. [14] have characterized three HMW NPs from the thermophiles *Deinococcus geothermalis*, *Geobacilus thermoglucosidasius* and *Aeropyrum pernix*. All these NPs catalyze both 6-oxo and 6-amino purine substrates and some analogs, although the natural substrates (inosine and adenosine) are better substrates than their 2-amino modified substrates. An intriguing discovery was the activity of these enzymes against cytidine; this activity (12–26 mU·mg^-1^) was several orders of magnitude lower than that observed for natural substrates (50–500 U·mg^-1^). The Nucleoside Phosphorylases can thus be thought as promiscuous enzymes involved in catalysis of one type of chemistry with many different substrates [15] (S1 Fig shows a 2D drawing of nucleosides and their bases for mnemonic purpose).

The structure and functional properties of the *S*. *mansoni* purine nucleoside phosphorylase isoform 1 (*Sm*PNP1) were reported by our group previously [16–24]. The enzyme uses inosine and guanosine as substrates as it has been determined that the main determinant specificity of LMW PNPs towards 6-oxopurines is due to the presence of asparagine in the active site (Asn243 in human and Asn245 in *S*. *mansoni* PNPs). Site-directed mutagenesis, replacing the asparagine to aspartic acid in human PNP leads to an increase of 5,000 fold of adenosine *k*_cat_ and a 4,000 fold increase in catalytic efficiency for adenosine [25].

Here, we characterize a newly identified isoform of *S*. *mansoni* PNP (*Sm*PNP2) employing a combination of methodologies: ligand discovery by thermofluorescence, kinetic assays, high-performance liquid chromatography (HPLC) and X-ray crystallography. Structural information of *Sm*PNP2 in both the apo form and in complexes with cytidine, cytosine, ribose-1-phosphate, adenine, hypoxanthine, and tubercidin was obtained.

These data allow us to describe, for the first time, the kinetics and structure of a LMW PNP well suited for inosine, adenosine and unexpectedly cytidine phosphorolysis. Finally, we also describe the structural basis for binding and catalysis of both purine and cytidine nucleosides.

## Material and methods

### Cloning, expression, and purification of recombinant *Sm*PNP2

The *Sm*PNP2 gene (Smp_179110) was identified in the *S*. *mansoni* genome [26]. The *Sm*PNP2 open reading frame (ORF) gene was synthesized with codon optimization by GenScript company and cloned into pOPINS3C [27] using the same protocol as previously described [28].

Two different protocols were employed for *Sm*PNP2 purification: the OPPF-UK protocol and the Physics Institute of Sao Carlos (IFSC-USP) protocol, the latter due to solubility problems faced with the first one. In OPPF-UK, the cells were defrosted and lysed in lysis buffer (50 mM Tris-HCl pH 7.5, 500 mM NaCl, 30 mM imidazole and 0.2% Tween-20) supplemented with 25 μL/mL of cocktail protease inhibitors (Sigma-Aldrich) and 400U/mL of DNAse I. Cells were lysed by Z cell disruptor at 30 kpsi and cell debris were removed by centrifugation (13,000 g for 45 min. at 4 °C). The clarified lysate was applied to a Ni2+-NTA column (GE, Healthcare) connected to an AKTA-Xpress purification system. Recombinant 6His+SUMO–*Sm*PNP2 was eluted with elution buffer (50 mM Tris pH 7.5, 500mM NaCl, 500mM imidazole) and injected into a size exclusion S200 column, pre-equilibrated with Gel filtration buffer (20 mM Tris pH 7.5, 200 mM NaCl and 1 mM Tris (2-carboxyethyl)phosphine (TCEP)). Fractions containing 6His+SUMO–*Sm*PNP2 were pooled and concentrated to 2–3 mg/mL.

In the second purification protocol, the cells were defrosted and lysed in a different Lysis buffer (50 mM potassium phosphate pH 7.4, 300 mM NaCl, 10 mM imidazole, 5 mM β mercaptoethanol, 0.1 mM dithiothreitol (DTT) and 1 mM phenylmethylsulfonyl fluoride (PMSF)). Cells were lysed by sonication followed by centrifugation (1,300 g for 45 min. at 4 °C). The clarified lysate was applied to Co-NTA agarose column (Qiagen), after column wash, the 6His+SUMO–*Sm*PNP2 was step-eluted with elution buffer (50 mM potassium phosphate pH 7.4, 300 mM NaCl, 200 mM imidazole and 5 mM β mercaptoethanol). Purified protein was visualized on a 12% SDS-PAGE after both purifications.

In both of the purification protocols, the 6His–SUMO tag was cleaved by addition of 3C protease (1 U/100 μg fusion protein) with overnight incubation at 4 °C. Cleaved *Sm*PNP2 was purified from the digest using a reverse purification in Ni2+-NTA column, the collected eluate was then concentrated and followed by dialysis in Tris-HCl buffer (20 mM Tris pH 7.4, 200 mM NaCl and 1 mM TCEP). Purified protein was concentrated up to 5–6 mg/mL in the appropriate buffer for further studies. All stages of *Sm*PNP2 production were visualized on a 12% SDS-PAGE.

### Differential scanning fluorimetry (DSF)

Employing the same procedures for activity assay used for *Sm*PNP1 [18], no activity for inosine phosphorolysis was observed for *Sm*PNP2. For this reason, DSF experiments were employed in order to identify a possible ligand for *Sm*PNP2.

Firstly, optimal concentrations of *Sm*PNP2 and SYPRO orange dye were determined to perform DSF on a grid of varying concentrations of *Sm*PNP2 protein and SYPRO orange in the Analysis Buffer (20 mM potassium phosphate pH 7.4, 300 mM NaCl). A BioRad CFX96 thermocycler was then programmed to equilibrate the sample at 25 °C for 5 minutes followed by an increase in temperature up to 95 °C, at a rate of 0.5 °C/min. The best condition found was 6 μM of *Sm*PNP2 and SYPRO orange 1:2000 dilution (dye: analysis buffer).

For ligand screening the Silver Bullets Bio kit (Hampton Research) was used. The reaction was performed using the best condition obtained as described above and contains 2 μL of Silver Bullets bio, in a final volume 20 μL. The apparent melting temperature (T_m_) was calculated from the mean of triplicate measurements and, the ΔT_m_ was calculated based on the difference of the T_m_ value from controls in absence of ligands and, the T_m_ value from the samples employing the DSF Melting Analysis (DMAN) software [29].

### Isothermal Titration Calorimetry

All of the ITC binding and kinetics experiments were carried out with an iTC200 microcalorimeter (GE Healthcare Lifesciences). Protein, ligands, and/or substrates were prepared in 50 mM potassium phosphate buffer (pH 7.6) plus 300 mM NaCl.

#### Binding experiments

In order to verify the potential of the cytosine base as a substrate for *Sm*PNP2, a preliminary binding experiment was performed. The sample cell was filled with 60 μM of protein (in the monomer concentration) and it was titrated with multiple injections of 1 mM cytosine, at 25 °C. The enthalpy for each injection was calculated by integrating the area under the peaks obtained from the change of power as a function of time. The heat of the injectant dilution was determined from the end of the titration and was subtracted from the data. The experimental isotherms were analyzed to yield an apparent association constant (*K*_A_), an apparent enthalpy change (Δ*H*_app_) and a stoichiometric coefficient (n). The apparent Gibbs energy change (Δ*G*_app_) was calculated using the relation Δ*G*_app_ = −*RT*ln*K*_A_ where *R* is the gas constant (cal.mol^-1^.K^-1^) and *T* the absolute temperature (K). The apparent entropy change (Δ*S*_app_) was estimated by the follow equation Δ*G*_app_ = Δ*H*_app_ − *T*Δ*S*_app_. The apparent dissociation constant (*K*_D_) was calculated as the inverse of the *K*_A_ value.

#### Kinetic experiments

Two different experiments were performed to determine the kinetic parameters of the cytidine nucleoside phosphorolysis by *Sm*PNP2. For determination of the apparent enthalpy change Δ*H*_app_ for the phosphorolysis, 13 μM *Sm*PNP2 was titrated with 3 injections of 5 μL cytidine nucleoside 5 mM, at 20 °C where the substrate was quickly and completely converted into product. The integral of the area under each peak yields the Δ*H*_app_ of the reaction. For determination of the rate of substrate reaction, 500 nM *Sm*PNP2 was titrated with 25 injections of 1 μL cytidine 5 mM, at 20 °C. After the correction for the heat of the titrant dilution, these data were analyzed using the Origin 7.0 to obtain values for *K*_M_, *V*_max_, and *k*_cat_ for the enzymatic reaction, as indicated by the supplier. The kinetic parameters were determined as the average values of the parameters obtained after analysis of three experiments.

### Adenosine and inosine phosphorolysis assays

Using an enzyme amount three-fold higher than previously described for *Sm*PNP1 assays [18], we were able to detect kinetic the parameters for adenosine and inosine. The kinetic parameters for adenosine were measured via a coupled assay by xanthine oxidase. In this method, the xanthine oxidase converts free adenine from adenosine (Ado) to 2,8-dihydroxyadenine, resulting in an increase in the absorbance at A_305_ nm (ε = 15,500 AU) [30,31]. The kinetic parameters for inosine were also measured using coupled assay by xanthine oxidase, which converts the hypoxanthine formed into uric acid, resulting in an increase in the absorbance at A_295_ nm (ε = 14,000 AU).

Kinetic parameters were calculated in sextuplicate (from three independent experiments) at room temperature in a 200 μL reaction mix containing 100 mM potassium phosphate buffer at pH 7.4, twelve concentrations of substrate (4.5 to 400 μM) and 0.3 units of xanthine oxidase from bovine milk (Sigma-Aldrich). The reaction was started by adding 1 μM *Sm*PNP2 to the reaction mixture, and the OD was immediately monitored using a SPECTRAmax^®^ PLUS384 spectrophotometer (Molecular Devices, USA). The kinetic parameters (*K*_M_ and *k*_cat_) were derived from non-linear least-squares fits of the Michaelis-Menten equation in the GraphPad Prism software using the experimental data.

### *Sm*PNP2 HPLC data analysis

The HPLC was used to identify the *Sm*PNP2 conversion of cytidine to cytosine in the presence and absence of phosphate. Firstly, the reaction was prepared in 50mM potassium phosphate buffer pH 7.4 with 1 mM cytidine and started with the addition of 200 nM *Sm*PNP2 at room temperature. Aliquots were withdrawn from time 0 to 40 minutes of the reaction (T_1_, T_2_, T_5_, T_10_, T_15_, T_20_, T_30_ up to T_40_, respectively), frozen in liquid nitrogen then heated at 85 °C for 15 minutes to denature the *Sm*PNP2, centrifugation at 10000 rpm for 20 minutes at 4 °C was used to remove the precipitated protein. The controls: phosphate buffer; cytidine; cytosine; ribose-1-phosphate (R1P) and a mix of all these substrates were prepared in the same buffer and treated to the same procedure.

An aliquot of 10 μL was applied to a Supelcosil^™^ LC-18-S (Sigma-Aldrich) column coupled to HPLC Water system with 1 mL/min flowrate and an isocratic gradient using 88% Buffer A (97.5 mM potassium phosphate buffer pH 4.0 and 2.5% methanol) and 12% Buffer B (97.5mM potassium phosphate buffer pH 4.0 and 25% methanol) monitored at 253 nm.

The reverse reaction using 1 mM cytosine in presence of 1 mM R1P was prepared in 50mM HEPES buffer pH 7.4 at room temperature. The enzymatic reaction was started with 200mM *Sm*PNP2 and monitored as previously mentioned and injected in the Supelcosil^™^ LC-18-S column to for analysis by HPLC.

### Crystallization, data collection, structure solution and refinement

Initially, a Pre-Crystallization Test (PCT) was performed (using *Sm*PNP2 in Tris buffer pH 7.5), to determine the optimal concentration of *Sm*PNP2 for setting up crystallization experiments. Using *Sm*PNP2 at a concentration of 5 mg/mL, crystallization screening experiments were performed on Cartesian 2 (OPPF-UK) using screen solutions from Molecular Dimension (Morpheus^®^ and JCSG-*plus*^™^), Hampton Research (Index HT^™^) and Jena Bioscience (JBScreen Wizard 1 and JBScreen Wizard 2) in Greiner Crystal-Quick 96 well-sitting drop plates (Hampton research, USA). Plates were incubated at 20 °C in FORMULATRIX imager (USA).

For the co-crystallization experiments the *Sm*PNP2 at 5 mg/mL was incubated with 5mM each ligand (R1P, inosine, hypoxanthine, cytidine, and cytosine). Crystallization trials using the Morpheus and JCSG-*plus* screens with 1:1 μL drops in Honeybee 961 robot in Greiner CrystalQuick plates at 18 °C were carried out. In all cases, cubic shape crystals appeared after two days and reached their maximum size (~150 μm) in three days.

The crystals used in soaking experiments were grown in 100 mM Bis-Tris pH 6.5, 200mM ammonium acetate and 30% PEG3350. Soaking was carried out in mother solution (2 μL) plus 0.5 μL of ligand (30 mM cytidine, 6 mM hypoxanthine, 50 mM tubercidin and 6 mM adenine) dissolved in 100% DMSO, for one 1 hour. The crystals were mounted in micro loops, cryo-protected with 20% glycerol or PEG200 (if necessary) and cooled in liquid nitrogen. Diffraction data were collected using synchrotron radiation at beamlines I04 and I04-1 at Diamond Light Source (DLS, Harwell, UK). These data were indexed, integrated and scaled using Xia2 [32–35].

The *Sm*PNP2 apoenzyme structure was solved by molecular replacement using the Phaser program [36], employing the *Sm*PNP1 isoform (PDB ID 3FB1) [24] which shares 61% identity as a search model. The remaining structures were also solved by molecular replacement, using one of the previously refined structure as a search model. The refinement was carried out using Phenix [37] and the model building was performed with COOT [38], using weighted 2Fo–Fc and Fo–Fc electron density maps. In all cases the behavior of *R* and *R*_free_ was used as the principal criterion for validating the refinement protocol and the stereochemical quality of the model was evaluated with Molprobity [39]. The coordinates and structure factors have been deposited into the Protein Data Bank (PDB) under the following codes: *Sm*PNP2–apo (5CXQ), *Sm*PNP2–MES complex (5CXS), *Sm*PNP2–cytosine complex (5KO5), *Sm*PNP2–cytosine–R1P complex (5KO6), *Sm*PNP2–tubercidin complex (5TBV), *Sm*PNP2–adenine complex (5TBS), *Sm*PNP2–cytidine complex (5TBT) and *Sm*PNP2–hypoxanthine complex (5TBU). The PDB validation files are visualized in S9 to S14 Figs.

### Whole-mount *in situ* hybridization (WISH)

To produce the antisense RNA probes, DNA templates corresponding to segments of 200–300 bp of *Sm*PNP1 (Smp_090520) and *Sm*PNP2 (Smp_179110) were amplified from adult worms cDNA, using the following primers: PNP1 forward 5'-TGTCGAAAGCGATTTGAAGC-3' and reverse 5'-TCATTTCAGCAAGTACACAAAGAGA-3' and PNP2 forward 5'-CCATGAAATAGTTACTCGTTCTAACAA-3' and reverse 5'- TGTGACCCGAAAAATTTGTAATG-3'. The primers were designed to comprise of untranslated regions (UTR) in order to avoid cross-reaction of the probe due to the sequence similarity. The amplicons were cloned into the pGEM-T-Easy vector (Promega), which contains both T7 and SP6 promoters; the orientation of the strands was verified by DNA sequencing. Transcription reactions to synthesize digoxigenin (DIG)-labelled RNA probes were performed using the Riboprobe kit (Promega) and the clones in pGEM-T as templates. The WISH protocol was carried out utilizing the *in situ* hybridization optimized conditions previously optimized for *S*. *mansoni* [40,41]. Briefly, formaldehyde fixed adult worms were partially digested with proteinase K, incubated overnight at 56 °C in hybridization buffer containing the DIG-RNA probes (1 μg/mL). After being extensively washed, the worms were blocked with 10% horse serum in MABT (100 mM maleic acid, 150 mM NaCl, 0.1% Tween-20, pH 7.5), incubated overnight at 4 °C with anti-digoxigenin-AP Fab fragments (Roche) at 1:2000 dilution and developed with a NBT/ BCIP solution (Roche). The control group was incubated in hybridization buffer with sense DIG-RNA probe. Images were acquired using an Olympus BX53 microscope.

## Results and discussion

### The *Sm*PNP2 sequence and expression profile are markedly distinct from that of *Sm*PNP1

**Fig 1:**
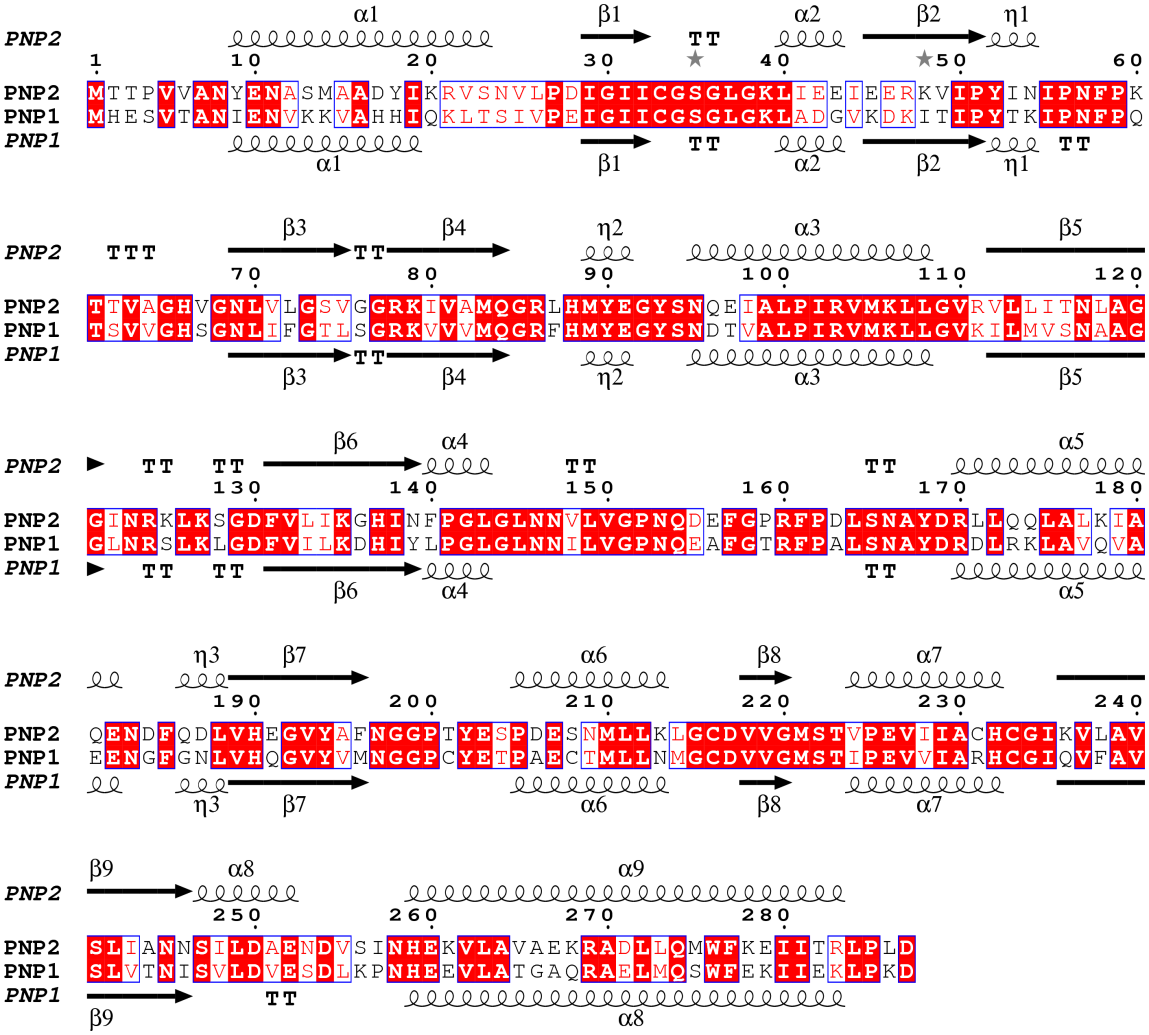
Alignment of *Sm*PNP1 and *Sm*PNP2 sequences. Highlighting the presence of an extra α-helix at residue 250 in *Sm*PNP2 structure. The sequences share 61% identity.

The sequence of *Sm*PNP2 was found searching the *S*. *mansoni* genome using GeneDB tools [42]. The sequence of *Sm*PNP2 encodes for a protein with 287 amino acids (the same size as *Sm*PNP1 protein), with a calculated molecular weight of ~31.3 kDa. The identity between the protein sequences of the two *S*. *mansoni* PNPs is 61% and *Sm*PNP2 has 46% identity to its human counterpart (Fig 1).

Comparison of expression profiles derived from previous RNA-Seq experiments [26] available at GeneDB showed that *Sm*PNP1 and *Sm*PNP2 present a different expression pattern: *Sm*PNP1 was highly expressed in adults while *Sm*PNP2 is more abundant in cercariae (with 24x higher transcription level than an adult worm). Moreover, WISH experiments using a probe specific for *Sm*PNP1 in adult worms resulted in a strong staining of female vitelaria (S2 Fig), indicating a specific role of this protein in the parasite reproduction and justifying its enrichment in adult worms. In contrast, WISH experiments using *Sm*PNP2 probes in adult worms were not successful, thus suggesting a low abundance of transcripts in this life stage. These data point out that *Sm*PNP1 and *Sm*PNP2 must be acting in very different and specific contexts and that these proteins may posess very distinct characteristics to fulfill their roles. Considering this scenario, a thorough investigation of the properties of *Sm*PNP2 was considered to be desirable to have further information about its possible role.

### *Sm*PNP2 is able to bind cytosine

The *Sm*PNP2 gene was synthesized with codon optimization and cloned in five pOPIN vectors [43]. After expression screening using several pOPIN vectors, we found that vector pOPIN-S3C, producing a fusion protein with SUMO, displayed best yields. After cleavage with PreScission protease, the *Sm*PNP2 was affinity purified using cobalt column. The yield was approximate 4 mg/L of culture medium.

Preliminary assays using *Sm*PNP2 showed very low activity against inosine (19 times lower than for *Sm*PNP1), which is a natural substrate for LMW PNPs. This unexpected characteristic of *Sm*PNP2 encouraged us to perform a DSF screening incubating the enzyme with mixtures containing several different compounds in order to find possible ligands.

An increase of T_m_ temperature was obtained in eight conditions varying from 2.5 to 0.5°C. Analyzing the composition of these mixtures that led to an increase in melting temperatures, we observed that with exception of one mixture (composed by L-carnitine, tannic acid, aspartame, caffeine, p-coumaric acid, 4-hydroxyl-proline and Hepes), all the remaining conditions contained cytidine or cytosine. Curiously, no significant T_m_ increase was observed when *Sm*PNP2 was incubated with mixtures containing purines, suggesting less binding with such compounds.

The lack of activity against inosine (in comparison to *Sm*PNP1 [19]) and the possible binding with cytidine and cytosine was completely unexpected. To our knowledge, no LMW PNP have been shown to have activity against pyrimidine bases or their nucleosides. Furthermore, in the canonical metabolic pathways cytidine is never directly converted to cytosine. Instead, it is either converted directly to cytidine monophosphate (CMP) by uridine–cytidine kinase (E.C 2.7.1.48) or is deaminated to uridine by cytidine deaminase (E.C 3.5.4.5).

Isothermal Titration Calorimetry using cytosine and *Sm*PNP2 confirmed binding displaying a stoichiometric coefficient of 1 base per protomer and a *K*_D_ equal to 27 (± 3) μM, the interaction being both enthalpic and entropy (Table 1 and S3 Fig).

**Table 1:** Thermodynamic parameters of the interaction between S*m*PNP2 and cytosine, obtained by Isothermal Titration Calorimetry.

Parameter	Value
Binding Sites (n)	0.71 ± 0.07
Δ*H*_app_ (cal/mol)	- 4700 ± 100
Δ*S*_app_ (cal/mol/deg)	+ 5.14 ± 0.06
*K*_A_ (M^-1^)	37000 ± 4000
*K*_D_ (μM)	27 ± 3.0

### *Sm*PNP2 efficiently catalyzes the phosphorolysis of cytidine

**Fig 2:**
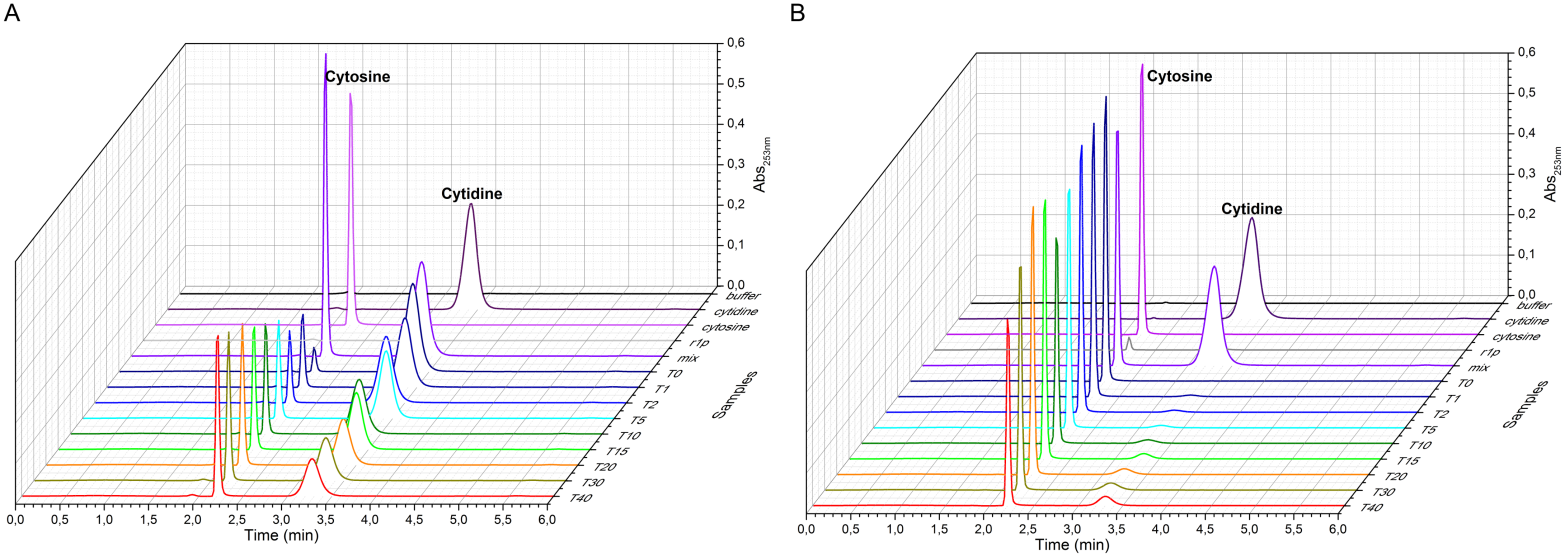
The HPLC chromatogram. **A**. Cytosine formation from cytidine in the presence of PPi measured by increase in absorbance at 253 nm at 2.3 min. **B**. The reverse reaction in HEPES buffer showing the inefficiency of *Sm*PNP2 for this enzymatic reaction by the maintenance of the absorbance for cytosine.

Considering the verified affinity of *Sm*PNP2 to cytosine, an obvious question is whether this protein has the capacity to catalyze the phosphorolysis of cytidine into cytosine. To address this, an HPLC analysis of a cytidine solution incubated with *Sm*PNP2 was performed, running aliquots corresponding to different times of incubation and monitoring the column eluate at 253nm to detect nucleotides. Comparison of retention times for cytidine and cytosine were determined using standards and allowed us to deduce an increase of cytosine concomitantly to a decrease in cytidine absorbance during the experiment time (Fig 2A). The reverse reaction was also prepared and analyzed using the same procedure and indicated a reduced efficiency in cytidine formation from cytosine in comparison with the rate observed for cytidine phosphorolysis (Fig 2B).

In addition, preliminary ITC binding assays of *Sm*PNP2 with cytidine showed that it was not possible to achieve saturation even after performing assays in several experimental conditions. Indeed, analysis of the titration showed a profile compatible with the catalysis of the titrator, thus providing further direct evidence of cytidine nucleoside phosphorolysis by *Sm*PNP2. The kinetics of enzyme-catalyzed reaction was obtained through two independent calorimetric experiments. In the first experiment, the Δ*H*_app_ was determined with high concentrations of enzyme in the cell, relatively low amounts of cytidine in the syringe and allowing sufficient time between the injections to ensure that all of the substrates were converted to product (S4 Fig, *inset*). The obtained peaks were used to deduce the Δ*H*_app_ value for the catalytic reaction. The same experiment performed for purine (adenosine, guanosine, and inosine) and pyrimidine (thymidine and uridine) nucleosides did not evolve heat exchange in the tested conditions (data not shown), since this absence does not prove the absence of reaction, to overpass this limitation we employed a well characterised spectophotometric assay.

In the second experiment, the rate data was obtained with low amounts of *Sm*PNP2 in the cell and high amount of cytidine nucleoside in the syringe, ensuring that steady-state conditions are maintained while substrate concentration is kept almost constant (S4 Fig). The data reaction obtained through the titration was fitted with the Michaelis-Menten model, allowing the calculation of the kinetic parameters (Table 2 and S4 Fig). The *K*_M_ value obtained for the cytidine phosphorolysis (76.3 ± 0.3 μM) represents an intermediate value when compared to the spectrophotometric assays for inosine (136 ± 7 μM) and adenosine (22 ± 2 μM), respectively. In the same way, parameters related to the catalytic efficiency as *k*_cat_ and *k*_cat_/*K*_M_ are also similar for cytidine, inosine and adenosine phosphorolysis (Table 2). These results are in contrast to the DSF experiment where no temperature increase was observed for purine nucleosides and bases, this may be due to the low concentration of purines in the assay.

**Table 2:** Kinetic parameters of the phosphorolysis promoted by S*m*PNP2.

Substrate	*K*_M_ (μM)	*k*_cat_ (s^-1^)	*V*_max_ (mM/s)	*k*_cat_/*K*_M_ (mM^-1^.s^-1^)
Adenosine	22.3 ± 2.5	1.02 ± 0.02	0.001	46.0 ± 5.2
Inosine	136.4 ± 7.0	7.0 ± 0.2	0.006	50.6 ± 2.9
Cytidine	76.3 ± 0.3	2.0 ± 0.2	0.001	26.2 ± 0.1

It should be noted that the only PNPs previously described to be capable of cytidine phosphorolysis displayed an activity several orders of magnitude lower than that observed for purines. In contrast, *Sm*PNP2 did not display a strong preference for purines in relation to cytidine and the relative concentration of nucleotides seems to be the determining factor. Therefore, it is possible argue that *Sm*PNP2 is the first PNP displaying a cytidine phosphorolysis activity in levels that might be relevant for cellular metabolism.

### *Sm*PNP2 crystal structures suggest different gate loop dynamics in the active site

In order to obtain further insights in relation to *Sm*PNP2 nucleoside specificity, the crystallographic structure of the protein was obtained with different ligands. *Sm*PNP2 was readily crystallized in several conditions, ranging from pH 4.0 to pH 9.0, in cubic space group P2_1_3 with one monomer per asymmetric unit. Most of the *Sm*PNP2 crystals from the co-crystallization experiments with cytidine show cytosine in the active site. Furthermore, a ternary complex with cytosine and R1P was also obtained, indicating the catalytic capability of *Sm*PNP2 in the crystallization solution and/or during incubation time. Soaking experiments using high ligand concentration was employed, resulting in four new *Sm*PNP2 complexes with adenine, hypoxanthine, tubercidin, and cytidine. These data collection and refinement statistics are presented in Table 3.

**Table 3:** Data collection, processing and refinement parameters.

	*Sm*PNP2-apo	*Sm*PNP2-MES	*Sm*PNP2-CYT	*Sm*PNP2-CYT-R1P	*Sm*PNP2-Adenine	*Sm*PNP2-Cytidine	*Sm*PNP2-Hypoxanthine	*Sm*PNP2-Tubercidin
Detector	Pilatus 2M	Pilatus 2M	Pilatus 6M	Pilatus 6M	Pilatus 6M	Pilatus 6M	Pilatus 6M	Pilatus 6M
Cell parameters (Å) a; b;c	99.55; 99.55; 99.55	97.78; 97.78; 97.78	99.13; 99.13; 99.13	98.64; 98.64; 98.64	100.83; 100.83; 100.83	100.14; 100.14; 100.14	99.70; 99.70; 99.70	91.98; 91.98; 91.98
Space Group	P2_1_3	P 2_1_3	P 2_1_3	P 2_1_3	P2_1_3	P 2_1_3	P 2_1_3	P 2_1_3
Resolution (Å)	44.52–1.57 (1.61–1.57)	43.73–1.74 (1.79–1.74)	70.10–1.36 (1.41–1.36)	69.75–1.42 (1.46–1.42)	71.30–1.90 (1.94–1.90)	57.82–2.10 (2.16–2.10)	70.00–2.10 (2.16–2.10)	65.04–1.95 (2.00–1.95)
X-ray Source	DLS I04-1	DLS I04-1	DLS I04	DLS I04	DLS I04-1	DLS I04-1	DLS I04-1	DLS I04-1
λ (Å)	0.920	0.920	0.979	0.979	0.920	0.920	0.920	0.920
Multiplicity	6.8 (7.0)	11.0 (10.5)	10.6 (10.0)	11.4 (9.1)	7.6 (7.8)	6.9 (7.0)	6.7 (4.7)	5.4 (5.3)
R_merge (%)_	5.5 (62.3)	5.8 (68.9)	13 (93.1)	7.6 (147.3)	7.0 (77.9)	18.2 (140.1)	15.4 (75.4)	8.7 (74.1)
R_pim_ (%)	2.5 (27.1)	1.9 (23.3)	4.2 (46.2)	2.4 (56.0)	2.9 (32.1)	7.9 (61.5)	6.9 (42.4)	4.5 (39.2)
CC(1/2)	0.998 (0.832)	1 (0.844)	0.994 (0.60)	0.999 (0.540)	1.000 (0.803)	0.994 (0.598)	0.993 (0.627)	0.998 (0.710)
Completeness (%)	100 (100)	100 (100)	100.0 (100.0)	100 (99.9)	100.0 (100.0)	99.9 (100.0)	99.9 (99.1)	100.0 (100.0)
Reflections	312485 (23781)	354427 (22974)	737563 (43532)	689682 (40358)	205704 (13435)	137829 (11377)	130953 (7589)	104475 (7301)
Unique Reflections	46089 (3379)	32239 (2370)	69723 (7274)	60417 (4438)	27201 (1720)	19849 (1614)	19582 (1610)	19223 (1368)
*I*/σ	18.0 (2.9)	25.4 (3.6)	8.4 (1.5)	15.9 (1.3)	18.7 (2.6)	7.2 (1.5)	9.1 (1.8)	9.4 (1.8)
Reflections used for Refinement	46050	31691	69526	60379	27154	19795	19553	19152
R(%)	18.53	15.0	17.46	16.19	16.00	19.04	18.87	17.87
R_free_(%)	21.24	19.11	19.16	17.85	19.68	22.60	22.75	20.24
N° of protein atoms	2180	2173	2541	2550	2174	2170	2170	2158
N° of ligant atoms	-	11	29	30	25	23	22	28
B (Å^2^)	18.79	17.33	15.64	17.93	26.01	20.00	20.92	28.20
Coordinate Error (ML based) (Å)	0.15	0.18	0.18	0.16	0.22	0.22	0.25	0.18
Phase error (°)	21.18	17.07	21.68	17.39	18.55	22.93	21.49	22.86
*Ramachandran Plot*								
Favored (%)	97.54	98.94	98.6	97.89	98.59	98.94	98.59	98.58
Allowed (%)	2.11	1.06	1.40	2.11	1.06	1.06	1.41	1.42
Outliers (%)	0.35	0.0	0.0	0.0	0.35	0.00	0.00	0.00
All-atom Clashscore	1.81	3.38	4.27	2.92	2.71	2.26	1.35	1.81
*RMSD from ideal geometry*								
r.m.s. bond lengths (Å)	0.007	0.018	0.005	0.006	1.205	0.604	0.002	0.010
r.m.s. bond angles (°)	1.088	1.694	0.872	0.953	0.015	0.002	0.524	1.068
*PDB ID*	5CXQ	5CXS	5KO5	5KO6	5TBS	5TBT	5TBU	5TBV

As expected, the overall fold of *Sm*PNP2 is the same observed for other low molecular weight NPs, with a root-mean-square deviation (RMSD) of 1.17 Å when compared by superposition with trimeric *Sm*PNP1 structures (PDB IDs 1TCV, 3FAZ, and 3E0Q). The superposition of *Sm*PNP2 complex structures does not show any remarkable difference (RMSD of 0.47 Å for trimers and 0.33 Å for monomers). A larger RMSD was observed for *Sm*PNP2–tubercidin complex (0.82 and 1.24 Å for monomer and trimer, respectively). The main difference between the *Sm*PNP1 and *Sm*PNP2 structures is the presence of α-helix formed by residues 247–252 in the latter, at the beginning of the gate loop (residues 244–260).

**Fig 3:**
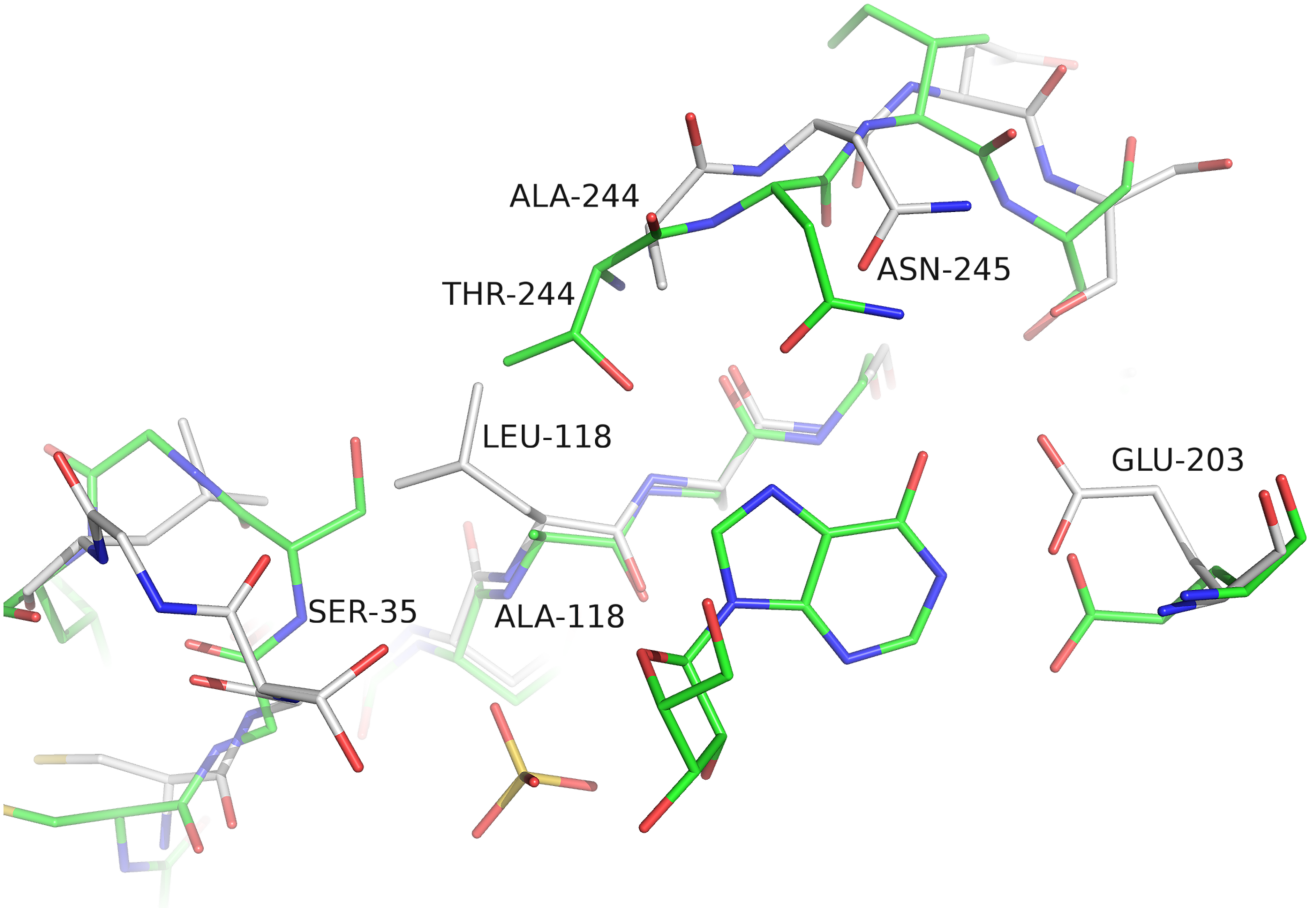
Comparison between *Sm*PNP1 (green backbone) and *Sm*PNP2 (white backbone) active sites. The presence of the Ala118Leu substitution results in the phosphate loop Ser35 displacement to an open position due to steric hindrance and thus the loop conformational change is no longer supported.

Comparison between both *Sm*PNPs isoforms reveals an intriguing substitution, of a residue (Ala118Leu; *Sm*PNP1:*Sm*PNP2) belonging both to the Phosphate Binding Site (PBS) and a highly conserved motif of the NP-1 family members forming the “*bottom*” of the active site [13]. This Ala118Leu substitution should have been preceded by a T244A substitution since a steric hindrance between side chains is predicted in a protein containing the T244 and an L118 (Fig 3). Erion et al [44], performed a site direct mutagenesis of Thr242Ala in human PNP, resulting in kinetic parameters for inosine and hypoxanthine similar to the wild-type enzyme, however, the *K*_M_ for phosphate is reduced 18 fold.

The presence of an Ala118Leu substitution results in conformational changes of the phosphate loop centered on the Ser35 residue. This loop, corresponding to residues 32–40 in *Sm*PNP1, can assume two different conformations (closed and opened). The presence of a bulkier leucine group in place of alanine next to the Ser35 residue in *Sm*PNP2 blocks the movement necessary to assume a closed conformation due to steric hindrance maintaining this loop permanently in an open conformation (Fig 3).

Another relevant substitution in the active site region is the Pro257Ile at the ending of the gate loop. The ring of Pro257 restricts the conformations assumed by Tyr202 (which forms a pi-stacking within the base). The presence of Ile257 displaces the residues Tyr202 and Glu203, and causes the reorientation of Asn245, reducing the volume of the active site cavity.

**Fig 4:**
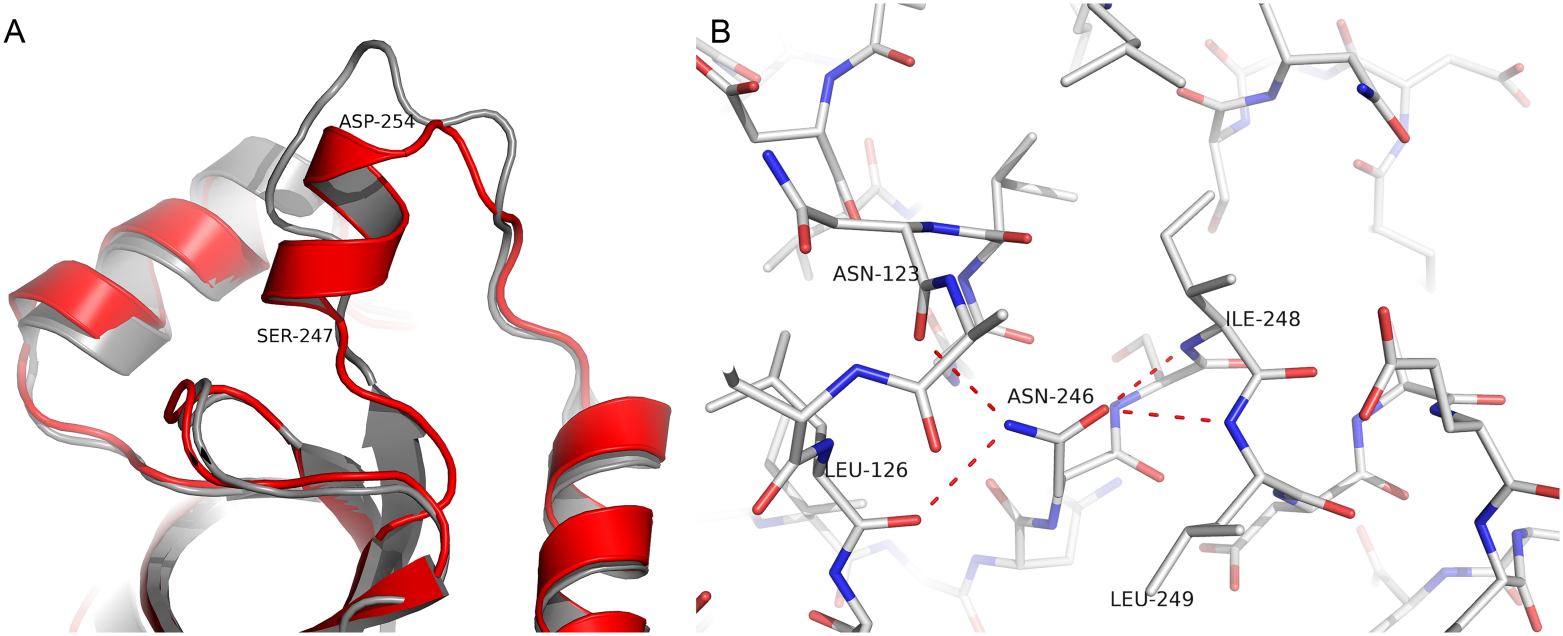
**A**. Ribbon model of *Sm*PNP1 (grey) and *Sm*PNP2 (red) superposition showing the extra α-helix formed by residues 247–252 in *Sm*PNP2. **B**. The formation of this α-helix is due to the presence of Asn246, which forms the N-terminus cap of the helix. The side chain of Asn246 forms 4 hydrogen bonds that lock this conformation.

**Fig 5:**
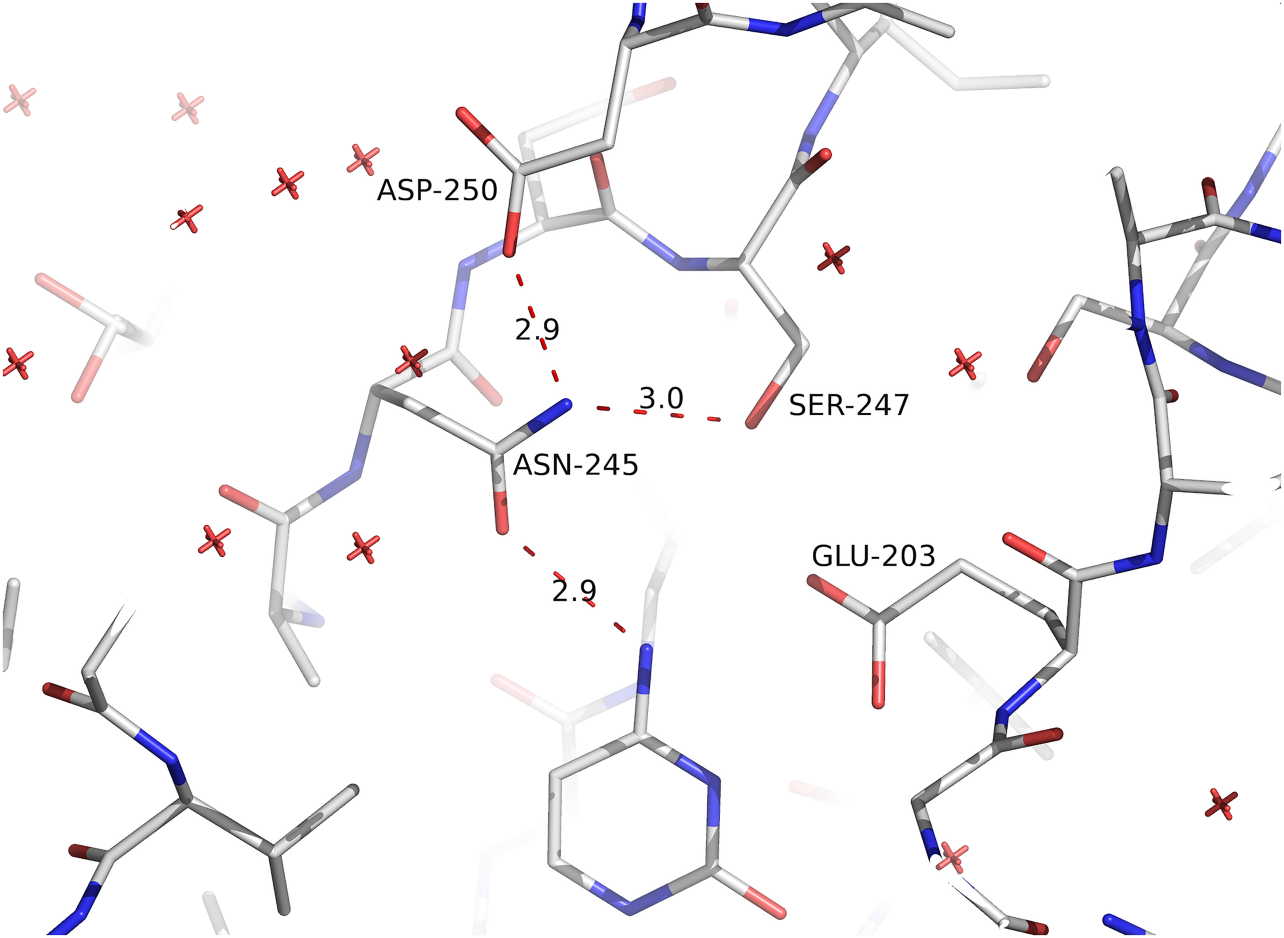
H-bond interaction between Asn245 and Asp250. This interaction helps Asn245 assume a non-canonical conformation, rendering *Sm*PNP2 unable to bind purine nucleosides as observed for *Sm*PNP1. This interaction is due to the α-helix-formation by residues 246–252.

**Fig 6:**
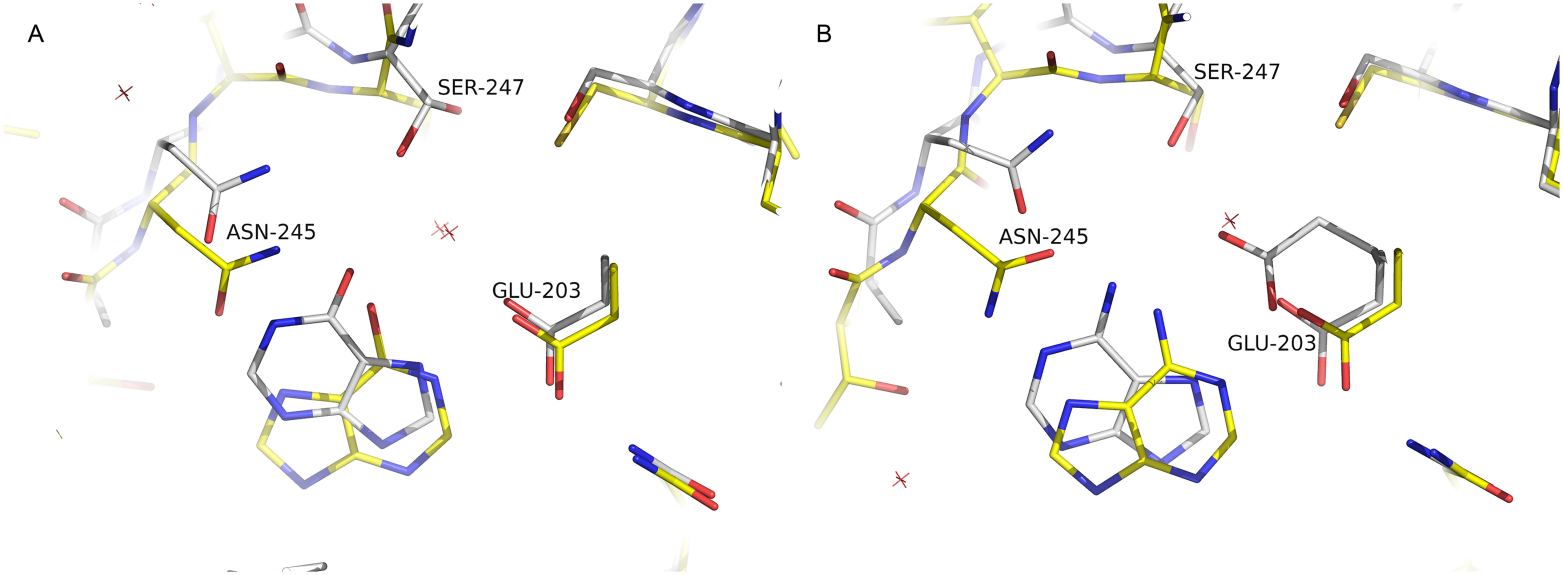
Purine bases in the *Sm*PNP2 active site show a very distinct orientation when compared to the *Sm*PNP1. **A**. Superposition of *Sm*PNP1 (yellow) and *Sm*PNP2 (white) hypoxanthine complexes. In *Sm*PNP2, hypoxanthine displays a completely different binding mode where it is flipped 180° compared with the canonical binding mode observed for *Sm*PNP1 and other LMW PNPs. The same binding mode is observed for adenine. **B**. Superposition of *Sm*PNP1 (yellow) *Sm*PNP2 (white) adenine complexes. As observed for hypoxanthine, adenine binding mode resembles the former and also is flipped 180° in comparison to the *Sm*PNP1–adenine complex.

The presence of a new α-helix formed by residues 247–252 and the substitution Ala118Leu also appear to have a large impact in the active site and gate loop conformations. This can be seen by calculating the RMSD of Cαs of residues 244–247, which display a values 2.84 Å, thus indicating a considerable deviation from the structure of *Sm*PNP1 (Fig 4A). This displacement of residues 244–247 brings residue Asp250 in the vicinity of Asn245, resulting in an H-bond formation between Asn245 ND2 and Asp250 OD2, locking Asn245 in the new conformation (Figs 3 and 5). Consequently, the Asn245 side chain is unable to bind purine nucleosides in the same manner of *Sm*PNP1, as shown by the hypoxanthine, adenine (Fig 6), and tubercidin (S5 Fig) complex structures (discussed below). The residue Asn246 (Ile246 in *Sm*PNP1) appears to be the determinant for the appearance of the new α-helix in *Sm*PNP2, with the main chain Asn246 O and the OD1 atoms forming three hydrogen bonds (H-bonds) with main chain N of residues 248, 249 and 250 thus forming the N-cap of the α-helix, the ND2 of Asn246 also interacts with Asn123 O and Leu126 O (Fig 4B).

### Adenine and hypoxanthine bind *Sm*PNP2 active site in an unusual orientation

Using a soaking approach, three complexes were obtained: *Sm*PNP2–tubercidin (7-deaza-adenosine), *Sm*PNP2–hypoxanthine and *Sm*PNP2–adenine (The composite omit maps of these ligands can be seen in S6 and S7 Figs, respectively). The interaction of *Sm*PNP2 with tubercidin in the ribose binding site (RBS) and PBS are similar to that observed for the *Sm*PNP1–adenosine complex. A notable exception is the distance of the base in respect to the Glu203 is greater than that observed in *Sm*PNP1 previous crystals [19], meaning that interaction between Glu203 and tubercidin in *Sm*PNP2 became water-mediated (Glu203 OE1—w142—Tub N6) (S5 Fig).

A totally different scenario is observed for the binding of adenine or hypoxanthine in the *Sm*PNP2, where the bound base displays a 180° rotation in the Y axes followed a small rotation in the Z axes resulting in different interactions within Base Binding Site (BBS) in comparison to PNP1 (Fig 6). A water molecule that interacts with Glu203, Ser247 and the base in *Sm*PNP1 was also observed in the *Sm*PNP2–hypoxanthine complex (S7 Fig). The binding of adenine is similar to the hypoxanthine, however, a clear double conformation is observed for Glu203 (one of them is the canonical one), in this configuration the side chain of Glu203 interacts only with N7 (canonical conformation) (S7 Fig).

Considering that tubercidin, which is an adenosine analog, display a canonical binding mode, it is possible to speculate that both adenosine and inosine should be also binding in a canonical position. The main reason for that is the steric constraints related to the presence of ribose group, which would argue against a similar conformation seen for adenine and hypoxanthine. This would mean that only after the glycoside bond cleavage the base could be free to rotate in the BBS and assume a non-canonical orientation.

### *Sm*PNP2 crystal structures confirm specific ligation of cytosine and cytidine to the active site

**Fig 7:**
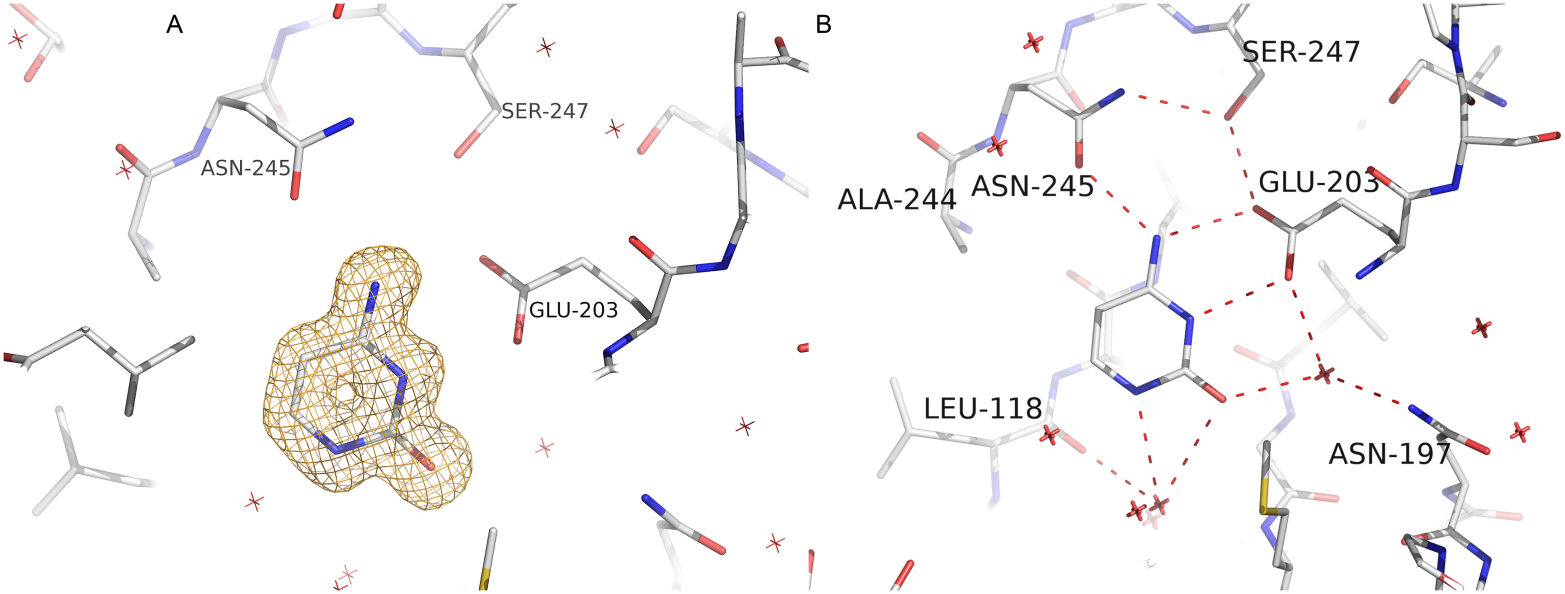
*Sm*PNP2 binds cytosine. **A**. Composite omit electron density map contoured at 1 σ for cytosine in the *Sm*PNP2 active site. **B**. Cytosine H-bond interactions network in the *Sm*PNP2 active site. The cytosine molecule forms 6 H-bonds with active site residues and water molecules (represented by a red X).

Analysis of the *Sm*PNP2–cytosine complex structure obtained at 1.36 Å places the cytosine molecule in the active site (Fig 7A), where it forms 6 H-bonds. The side chain of key active site residues Glu203 and Asn245 forms two and one H-bonds, respectively, and other three water-mediated bonds were found with residues Leu118, Asn197, and Met221 (Fig 7B). The conformation of the BBS is the same observed for *Sm*PNP2–apo structure, and both structures are essentially the same.

The ternary *Sm*PNP2–cytosine–R1P complex was also obtained at high resolution (1.42 Å) and it is well superimposed to *Sm*PNP2–cytosine structure with RMSD of 0.19 Å. Both cytidine and R1P do not present a sharp electron density map as expected for this resolution, indicating movement of these ligands in the active site and/or the presence of cytidine and cytosine/R1P at the same time in the crystal. This is true especially for the ribose moiety of the cytidine and for R1P (S8 Fig).

The binding of cytosine and R1P causes small rearrangements in the active site when compared with the *Sm*PNP2–cytosine complex. A small displacement of the Glu203 side chain occurs, increasing the H-bond distance (0.3 and 0.4 Å increase) with the cytosine resulting in a weak interaction. The interaction with Asn245 is maintained unaffected. As expected the R1P binds in *Sm*PNP2 in a similar way to that observed for *Sm*PNP1–R1P complex [24].

**Fig 8:**
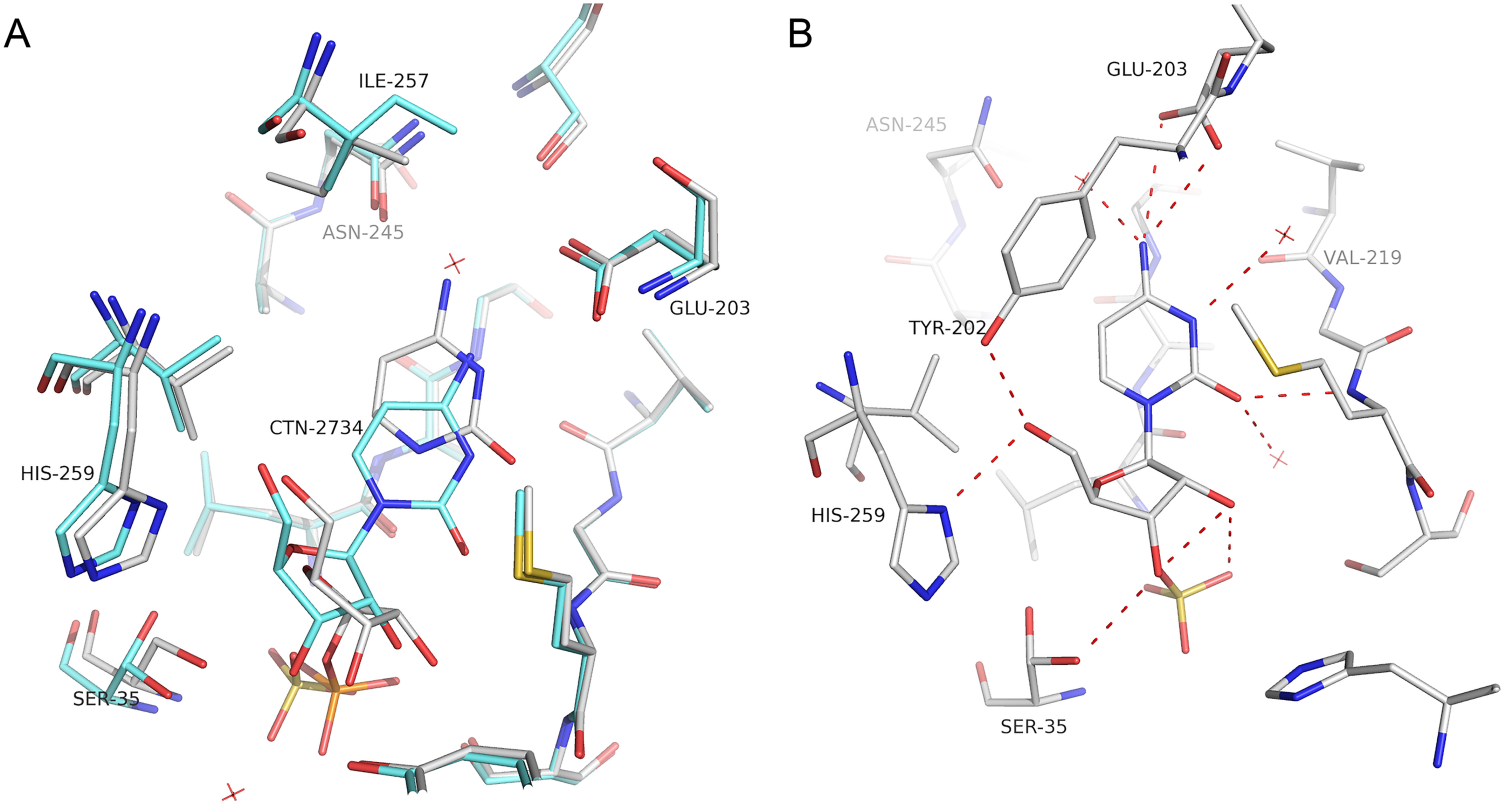
**A**. Superposition of cytidine (blue) and cytosine–R1P (white) *Sm*PNP2 complexes. **B**. Cytidine H-bond interactions in the *Sm*PNP2 active site. In contrast to the other nucleosides, no direct interaction was formed between the base and Asn245, the interaction is now mediated by a water molecule (represented by a red X).

A more noteworthy modification in the binding site is observed for the *Sm*PNP2–cytidine complex. Comparison with both *Sm*PNP2–cytidine and—cytosine–R1P complexes reveals that RMSD between cytosine moieties in both complexes is 1.73 Å (Fig 8A).

Interaction between *Sm*PNP2 and the cytosine moiety in cytidine occur through three direct H-bonds with residues Met221 (one) and Glu203 (two), and three water-mediated anchored by residues Glu203 and Asn245 (water 3), Glu203 and Asn197 (water 29), and Met221, Leu118, Tyr194 and Ser222 (water 45), this later also interacts with sulphate group which lies in phosphate binding site (Fig 8B).

The position of the ribose moiety in *Sm*PNP2–cytidine complex is displaced in comparison to what is observed in other *Sm*PNP1 and *Sm*PNP2 structures. Consequently, several side groups are moved to accommodate this new position for the ribose. The His259 ND1 and Tyr202 OH are both displaced when compared with the *Sm*PNP2–cytosine–R1P to form H-bonds with ribose O5. Small movements are also observed for the phosphate Ser35 loop and for His88 side chain. Moreover, a structural water that typically mediates the interaction between Tyr194 and the ribose O2’ interacts with cytidine O2 in *Sm*PNP2–cytidine complex and the canonical contacts with Met221 and Tyr90 are lost in this complex (Fig 8B).

## Discussion

**Fig 9:**
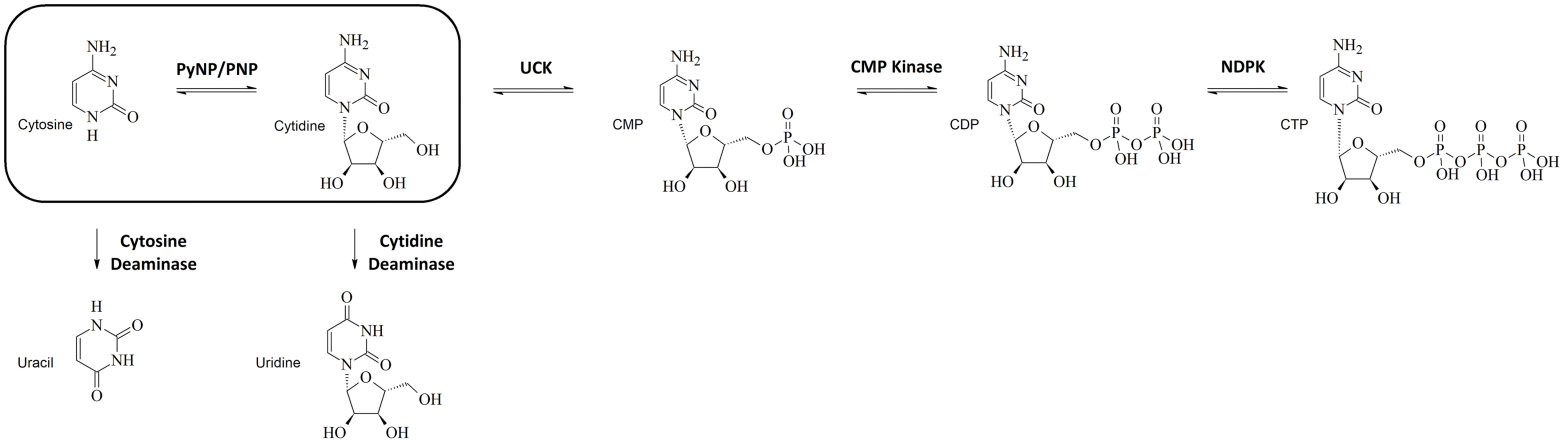
Section of the pyrimidine pathway for the cytosine to CTP conversion in *Schistosoma mansoni*. Pathway of Highlighted *Sm*PNP2 activity, that is able to convert cytidine into cytosine, this activity has never been described before to LMW NPs.

The unique properties of *Sm*PNP2 were discovered using a combination of techniques such as DSF, kinetics and X-ray crystallography. It is noteworthy that the ability of *Sm*PNP2 to catalyze the phosphorolysis of cytidine with similar efficiency to inosine represents a unique trait in a nucleoside phosphorylase (Fig 9). The crystal structure reveals this property appears to be related to the substitutions in the active site that induce the locking of the gate loop. Indeed, the mobility of active site loops has been ascribed to play a key role in mediating enzyme promiscuity [45], providing further support to this hypothesis.

Crystal structures of *Sm*PNP2 may also help to contribute to the understanding of catalysis in PNPs since different and sometimes contrasting catalytic mechanism has been proposed for phosphorolysis in LMW NPs [46]. For example, mechanisms that involve the protonation or stabilization of N7 from the purine ring promoted by An245 (Asn243, in human) [47,48] are not expected to occur within the structural framework observed in *Sm*PNP2, since this residue is now locked in new conformation and therefore a H-bond between Asn245 side chain and N7 is absent.

The fact that *Sm*PNP2 is capable of hydrolyzing adenosine and inosine with similar efficiency also exclude a mechanism proposed for HMW PNPs based on transition state where Glu204 (Glu201 in human PNP) forms a H-bond with exocyclic negatively charged O6 of rare enol purine tautomer [49]. This is because in such model adenosine could not be used as a substrate since no stabilization is possible with a negative charge at the base via an enolate intermediate and the lack of hydrogen at N1 of the base.

In contrast, an alternative mechanism proposed for HMW PNPs based on the formation of a negative charge delocalized to the base six-membered ring during transition state and a network of water molecules that connect N1 and O6 of the purine base [50], seems compatible with the *Sm*PNP2 structures. This is because that mechanism allows catalysis of both 6-oxo and 6-amino purines and *Sm*PNP2 has a water molecule anchored by residues Glu203 and Ser247, which would help in the stabilization of a charged intermediate, together with Glu201 side chain.

Moreover, a proposed catalytic mechanism for *Trypanosoma cruzi* uridine phosphorylase [51] seems an adequate mechanism to describe the mechanism for cytidine cleavage by *Sm*PNP2. Under the original model a protonated group, possibly uridine NH3, is essential for catalysis, its deprotonation generates a dianionic uracil a less effective leaving group. In the context of the catalysis promoted by *Sm*PNP2, we can assume that an analogous configuration could occur with Glu203 forming two H-bonds with cytidine N4 and one water molecule also binding N4 anchored by residues Glu201 and Asn245. In this context, a protonation of N3 could be achieved helped by a water molecule anchored by residue Asn197.

Analysis of Lu and co-workers [52,53] data related to gonad-specific and pairing-dependent transcriptome reveal that *Sm*PNP1 is approximately 160 times more expressed than *Sm*PNP2 in mature adult females. This is probably related to the fact that *Sm*PNP1 display high expression in female vitelaria, as revealed by our WISH experiments. Moreover, *Sm*PNP2 is significantly enriched in male’s testis when compared to its own expression in whole adult males (~25 times higher expression). This data suggest that both PNPs might have an important role in functions related with sexual tissue metabolism.

A possible explanation of *Sm*PNPs relative promiscuity in relation to the bound nucleotide could be related to scavenging of nutrients [15]. Cytidine is the pyrimidine nucleoside with the highest incorporation in adult worms (210 pM per 10 worms pairs) [11] and is used by the uridine-cytidine kinase to form CMP in the worm metabolism. In cercariae, where *Sm*PNP2 has the highest transcript levels, this enzyme could be involved in the production of R1P from nucleosides that could be subsequently used by to form Phosphoribosyl pyrophosphate (PRPP) in order to conserve nucleosides.

These structures and kinetics of *Sm*PNP2 provides more information about the nucleotide metabolism in general and in particular for *S*. *mansoni*, which enrich the understanding of the utilization of nucleotides for this important neglected parasite.

## Supporting information

S1 Fig 2D structures of nucleosides and bases. **A**. Inosine; **B**. Adenosine; **C**. Uridine; **D**. Cytidine; **E**. Hypoxanthine; **F**. Adenine; **G**. Uracil and **H**. Cytosine.
(PSD)

S2 Fig Localization of PNP1 transcripts in *S*. *mansoni* adult worms by WISH. PNP1 expression sites in male (A-B) and female (C-D) adult worms. B and D are higher magnification views of the boxed images in A and C, respectively. V, vitellaria of female worms.
(TIF)

S3 Fig ITC binding curve of *Sm*PNP2 and cytosine base. Top panel: Titration curve for 60μM *Sm*PNP2 with 1 mM cytosine at 25 °C. Thermodynamic parameters were derived from non-linear least-squares fitting. Bottom Panel: fit of the binding isotherm to the one set of sites model.
(TIF)

S4 Fig **A**. Multiple-injection titration of *Sm*PNP2, 13 μM, with 5 mM cytosine; **B**. Fit of the Michaelis-Menten model (solid line) to the *Sm*PNP2 protein reaction rate as a function of the added cytosine derived from the ITC data. *Inset*: Apparent enthalpy change experiment determination for the catalytic reaction.
(PSD)

S5 Fig Tubercidin binding in the *Sm*PNP2 active site. **A**. Tubercidin interactions in *Sm*PNP2 active site. In contrast to adenosine binding in *Sm*PNP1, no direct interaction was formed between Glu203 side chain and tubercidin. **B**. Superposition of *Sm*PNP1–adenosine (yellow) and *Sm*PNP2–tubercidin (white), show that with the exception of the different conformers for Asn245 side chain no other differences were observed, notably in this complex Glu203 assumes the canonical conformation.
(PSD)

S6 Fig Composite omit map countered at 1.0 σ for tubercidin in *Sm*PNP2 active site. (PSD)

S7 Fig **A**. Composite omit map countered at 1.0 σ for the *Sm*PNP2–hypoxanthine complex. **B**. Hypoxanthine H-bond interaction formed in the *Sm*PNP2 active site. **C**. Composite omit map countered at 1.0 σ for adenine. **D**. Adenine H-bond interactions in the *Sm*PNP2 active site.
(PSD)

S8 Fig **A**. Composite omit map countered at 1.0 σ for *Sm*PNP2–cytidine. **B**. Composite omit map countered at 1.0 σ for *Sm*PNP2–cytosine–R1P complex.
(PSD)

S9 Fig Validation report for PDB file 5tbt. (PDF)

S10 Fig Validation report for PDB file 5ko6. (PDF)

S11 Fig Validation report for PDB file 5ko5. (PDF)

S12 Fig Validation report for PDB file 5cxs. (PDF)

S13 Fig Validation report for PDB file 5cxq. (PDF)

S14 Fig Validation report for PDB file 5tbu. (PDF)
