# Supplementary figures and images for: The molecular structure of Schistosoma mansoni PNP isoform 2 provides insights into the nucleoside selectivity of PNPs
Source: PLoS One. 2018 Sep 7;13(9):e0203532. doi: 10.1371/journal.pone.0203532 (PMC6128611; doi:10.1371/journal.pone.0203532)

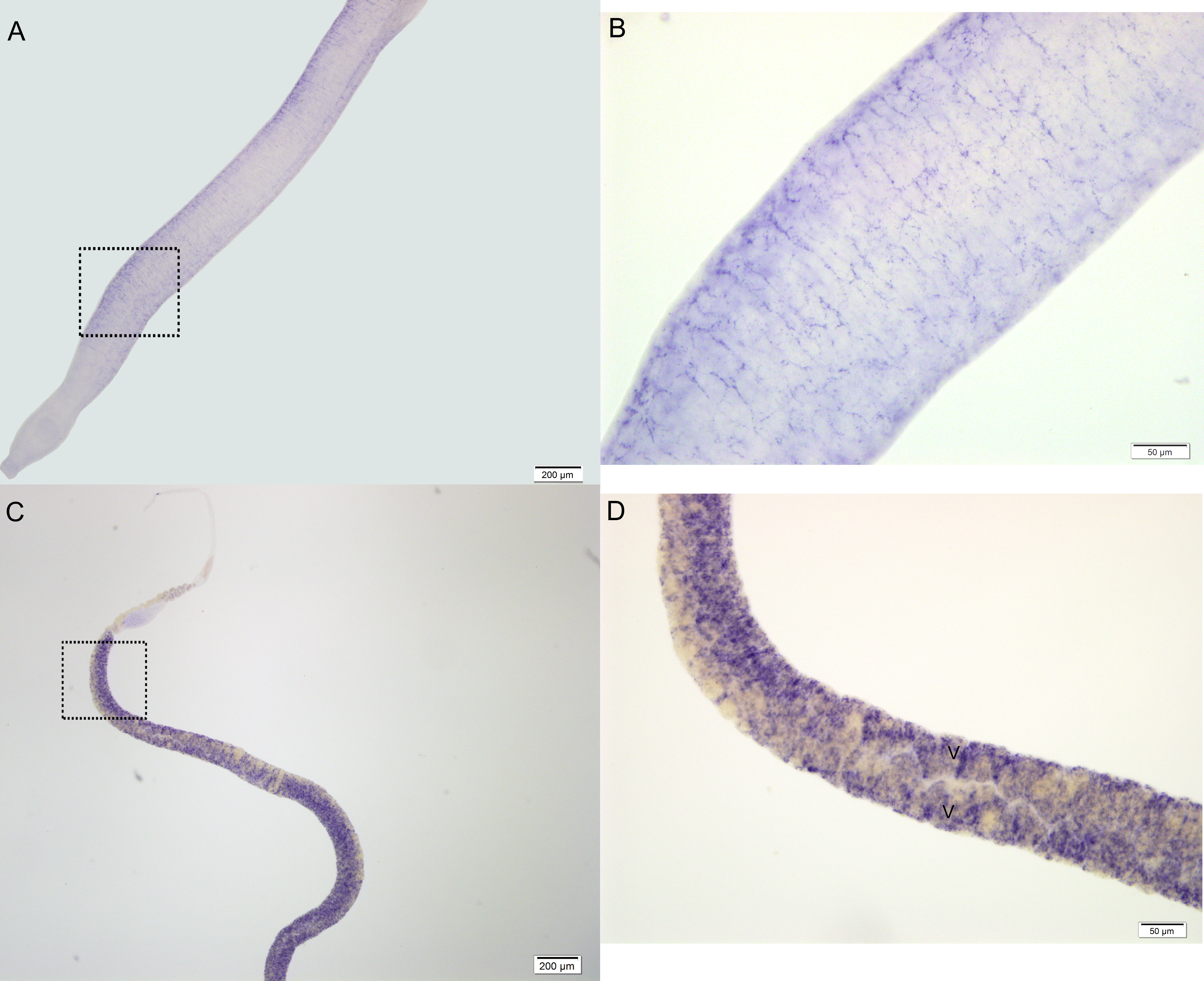

Supplement: S2 Fig — PNP1 expression sites in male (A-B) and female (C-D) adult worms. B and D are higher magnification views of the boxed images in A and C, respectively. V, vitellaria of female worms. (TIF) [file pone.0203532.s002.tif]
